# Concurrent validity of machine learning-classified functional upper extremity use from accelerometry in chronic stroke

**DOI:** 10.3389/fphys.2023.1116878

**Published:** 2023-03-22

**Authors:** Shashwati Geed, Megan L. Grainger, Abigail Mitchell, Cassidy C. Anderson, Henrike L. Schmaulfuss, Seraphina A. Culp, Eilis R. McCormick, Maureen R. McGarry, Mystee N. Delgado, Allysa D. Noccioli, Julia Shelepov, Alexander W. Dromerick, Peter S. Lum

**Affiliations:** ^1^ Department of Rehabilitation Medicine, Georgetown University, Washington, DC, United States; ^2^ MedStar National Rehabilitation Hospital, Washington, DC, United States; ^3^ Department of Biomedical Engineering, The Catholic University of America, Washington, DC, United States

**Keywords:** accelerometry, stroke rehabilitation, psychometrics, paresis/rehabilitation, sensors, disability evaluation, machine learning, ADLs

## Abstract

**Objective:** This study aims to investigate the validity of machine learning-derived amount of real-world functional upper extremity (UE) use in individuals with stroke. We hypothesized that machine learning classification of wrist-worn accelerometry will be as accurate as frame-by-frame video labeling (ground truth). A second objective was to validate the machine learning classification against measures of impairment, function, dexterity, and self-reported UE use.

**Design:** Cross-sectional and convenience sampling.

**Setting:** Outpatient rehabilitation.

**Participants:** Individuals (>18 years) with neuroimaging-confirmed ischemic or hemorrhagic stroke >6-months prior (*n* = 31) with persistent impairment of the hemiparetic arm and upper extremity Fugl-Meyer (UEFM) score = 12–57.

**Methods:** Participants wore an accelerometer on each arm and were video recorded while completing an “activity script” comprising activities and instrumental activities of daily living in a simulated apartment in outpatient rehabilitation. The video was annotated to determine the ground-truth amount of functional UE use.

**Main outcome measures:** The amount of real-world UE use was estimated using a random forest classifier trained on the accelerometry data. UE motor function was measured with the Action Research Arm Test (ARAT), UEFM, and nine-hole peg test (9HPT). The amount of real-world UE use was measured using the Motor Activity Log (MAL).

**Results:** The machine learning estimated use ratio was significantly correlated with the use ratio derived from video annotation, ARAT, UEFM, 9HPT, and to a lesser extent, MAL. Bland–Altman plots showed excellent agreement between use ratios calculated from video-annotated and machine-learning classification. Factor analysis showed that machine learning use ratios capture the same construct as ARAT, UEFM, 9HPT, and MAL and explain 83% of the variance in UE motor performance.

**Conclusion:** Our machine learning approach provides a valid measure of functional UE use. The accuracy, validity, and small footprint of this machine learning approach makes it feasible for measurement of UE recovery in stroke rehabilitation trials.

## 1 Introduction

The cornerstone of rehabilitation effectiveness lies in the answer to “how much did the individual use their affected upper extremity (UE) during functional activities in their environment?” Stroke rehabilitation trialists evaluate UE motor performance using clinical scales such as the Action Research Arm Test (ARAT) (Stinear et al., 2020) or self-reports of spontaneous UE use such as the Motor Activity Log (MAL) (Uswatte et al., 2006b). However, there are contextual differences between real-world UE use and UE motor performance in the clinic, completed following precise instructions as in the ARAT. In-clinic performance (capacity) does not always translate well to real-world use (performance) (Lundquist et al., 2022). The MAL suffers from biases associated with self-report scales (Prince et al., 2008) and disordered item difficulties that make the use of summed MAL scores a problem in measuring a meaningful change in clinical trials (van der Lee et al., 2004; Chuang et al., 2017). Furthermore, correlations between accelerometry, using the count thresholding method to quantify the amount of UE use, and the MAL are 0.52 (Uswatte et al., 2006a). Correlations between the ARAT and the MAL are reported to be 0.6 (van der Lee et al., 2004). Thus, the MAL has only fair to moderate validity and sensitivity for measuring real-world UE use (Uswatte et al., 2006b; Hammer and Lindmark, 2010). These limitations in clinical and self-report scales emphasize the need for alternative methods of directly measuring real-world functional use of the extremities.

Accelerometry is portable, unobtrusive, and suitable for 24/7 monitoring of patient activity. In the present report, to better quantify functional UE use in the community, we have advanced current accelerometry methods by validating a machine learning approach to classify UE movement as “functional” or “non-functional” in individuals with persistent motor impairment due to stroke at least 6 months prior (McLeod et al., 2016; Bochniewicz et al., 2017; Lum et al., 2020). Conventionally, quantifying UE use requires frame-by-frame video labeling for ground-truth validation. Video labeling, although ideal, is tedious and time-consuming, which makes it impractical for extended periods of home monitoring. Our machine learning approach identifies the amount of functional UE use in accelerometry data (test set) based on features of meaningful UE use extracted from a training dataset (labeled frame-by-frame using video ground truth). This advancement allows the estimation of functional UE use instead of just movement counts using accelerometry. In the present report, our purpose was to establish the concurrent validity of our machine learning estimate of functional UE use with respect to clinical measures of UE motor function (ARAT) and self-reported UE use (MAL). We hypothesized that machine learning-estimated characterization of real-world UE use will show significantly high correlation (*r* > 0.7) with ARAT and self-reported UE use. In a subset of the sample, we also validated our machine learning estimates with clinical measures of impairment (UEFM) and manual dexterity (nine-hole peg test) (Reuben et al., 2013; Wang et al., 2015).

## 2 Materials and methods

### 2.1 Participants

Individuals were recruited from the MedStar National Rehabilitation Hospital in Washington, DC. The inclusion criteria were 1) neuroimaging-confirmed ischemic or hemorrhagic stroke at least 6 months prior to study enrollment, 2) age >18 years old, 3) no known orthopedic or neuromuscular injuries that interfered with completion of study procedures, and 4) Mini-Mental Status Examination score >24 (Cumming et al., 2013). Individuals were excluded if 1) they exhibited neglect as determined by an asymmetry >3 errors on the Mesulam’s symbol cancellation test (Mesulam, 1985), 2) experienced dense sensory loss (NIHSS sensory item score ≥2) (Brott et al., 1989), 3) had prior stroke with persistent motor impairments, and 4) received botulinum toxin within 6 months of stroke or during study participation. The study was approved by the MedStar-Georgetown Universities Institutional Ethics Committee. All individuals provided written informed consent.

### 2.2 Power and sample size considerations

Sample sizes were calculated using the software program G*Power (Faul et al., 2007) to test if use ratios were significantly correlated with the Action Research Arm Test scale to demonstrate concurrent validity. We used a moderate effect size (0.4), power = 0.8, and alpha = 0.05/2, leading to a required sample size of approximately 17 participants. For a power of 0.95 at alpha = 0.05/2, we would have required a sample of approximately 30 participants. We report results from a cohort of 31 stroke patients with a wide range of UE motor impairments measured by ARAT.

### 2.3 Apparatus and measures

#### 2.3.1 Clinical testing

Data were collected over a single session when participants completed the activity script (Lum et al., 2020) and tests of UE motor function (ARAT) (van der Lee et al., 2001; Yozbatiran et al., 2008) impairment *via* UEFM (Fugl-Meyer et al., 1975; Weerdt and Harrison, 1985; Duncan et al., 1992) and manual dexterity (nine-hole peg test) (Wang et al., 2011; Reuben et al., 2013; Wang et al., 2015). Participants also completed the Motor Activity Log(MAL), a self-reported outcome of “how much” the impaired UE was used in the previous 7 days (Uswatte et al., 2006b). Ten out of 31 participants completed only the activity script and the ARAT, whereas the remaining participants completed all tests. Data from these 10 participants were part of a prior publication (Lum et al., 2020).

#### 2.3.2 Accelerometers

Wireless accelerometers (ActiGraph GT9X Link, Pensacola, FL), similar in appearance to a smartwatch, were worn on both wrists. The accelerometers are sensitive to movement in three axes, and raw acceleration is sampled and stored internally at 50 Hz.

### 2.4 Procedures

**FIGURE 1:**
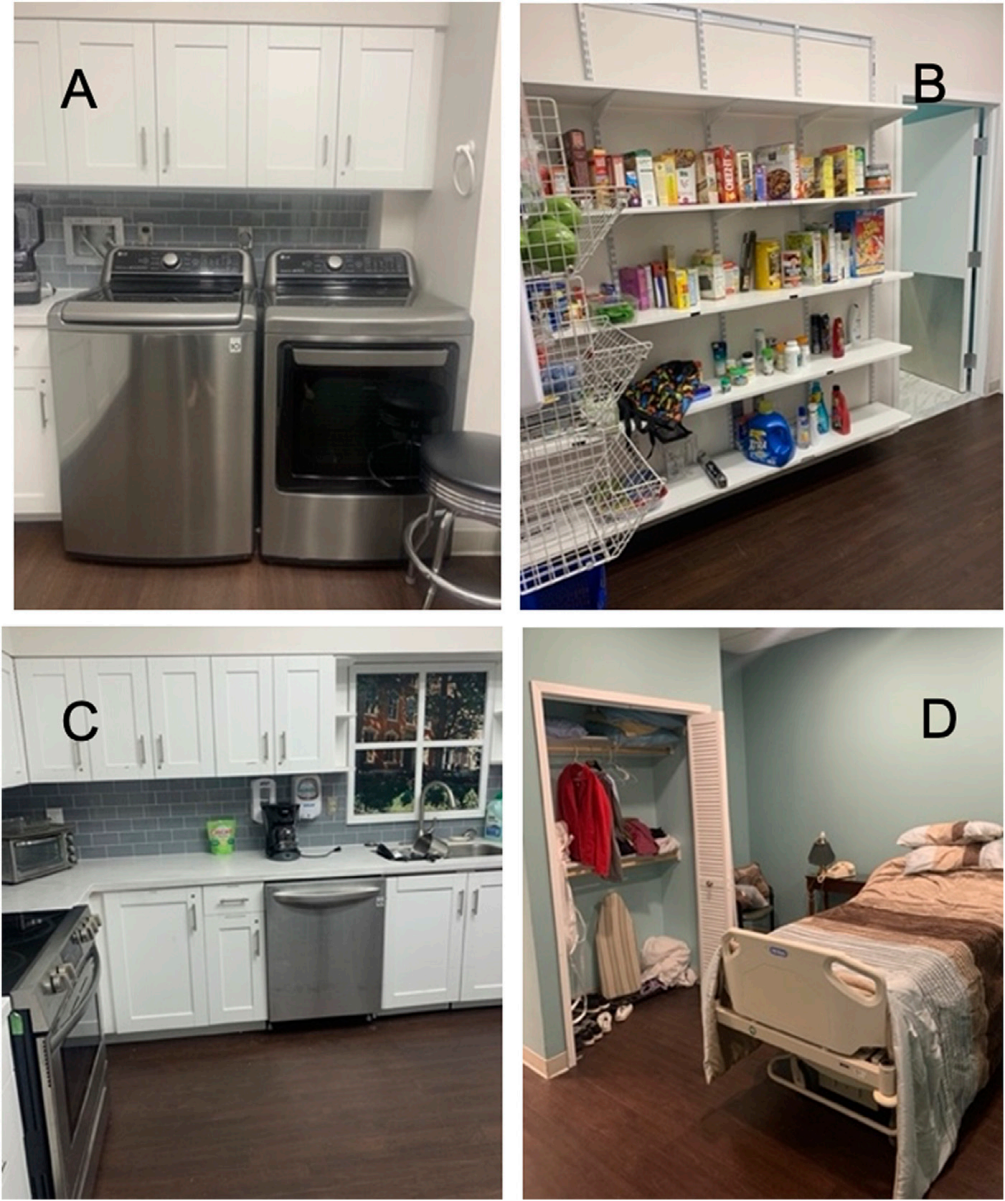
Simulated apartment in outpatient rehabilitation. **(A)** Laundry task. **(B)** Shopping task. **(C)** Kitchen task. **(D)** Bed-making and laundry-folding tasks. Activity scripts are completed using the facilities at MedStar National Rehabilitation Hospital, Washington DC.

Activity script: all participants completed the activity script, a set of activities and instrumental activities of daily living (ADLs/IADLs), to simulate functional UE use in the community. These procedures have been described previously (Lum et al., 2020). The activity script was completed in a simulated apartment in an outpatient rehabilitation setting (Figure 1). The simulated apartment houses a fully functional “living space,” including a kitchen, bedroom, store for shopping activities, and a car to practice transfers. Individuals were instructed to perform the following IADLs: 1) laundry task, 2) linen management and folding, 3) grocery shopping, 4) kitchen task, 5) financial management, 6) medication management, and 7) typing task (see Supplementary Table S1).

Participants performed the activity script tasks as they would naturally complete them at home. No specific instructions were given as to which arm to use for any task. Between the activity script tasks, when participants sat, experimenters engaged them in conversation, and they walked around the facility to collect non-functional UE use data. There was no set time limit to complete the activity script. Participants wore the sensors throughout the experiment and were videotaped at 30 Hz.

### 2.5 Data processing

#### 2.5.1 Video annotation

The video was annotated by trained research assistants using a standardized coding scheme based on the Functional Arm Activity Behavioral Observation System (FAABOS) (Uswatte and Hobbs Qadri, 2009). Three independent annotators watched the video. All frames were labeled according to the five FAABOS categories and subsequently collapsed into three categories: functional, non-functional, or unknown. The functional category included gesturing, reaching to grasp, pushing open a door, etc. The non-functional category included arm movements associated with gait, sit-to-stand, or whole-body movement that did not include functional arm movement. No movement was labeled non-functional. Each limb was coded separately, and the final coding for each video frame was determined by a majority vote. The final coding values allowed calculation of %functional use ratio: %functional use in the paretic limb normalized to the less-affected limb. Video-annotated use ratios are referred to as ground-truth use ratios.

#### 2.5.2 Application of machine learning algorithms to accelerometer data

The video was synchronized with accelerometry data, and ground-truth labels of functional or non-functional activity were transferred to the accelerometry data. Synchronization of accelerometry and video was achieved by oscillating the accelerometers rapidly in the *z* direction of the sensors five times prior to placing them on the subject and again after their removal. This created five large peaks in the *z*-axis data that were easily identified and marked. The sensor peaks correspond to reversal points in the oscillation, which were marked on the video. Data were partitioned into 2-second blocks. If >90% of labels within the block were the same class (functional or non-functional), the entire block was labeled accordingly. The remainder of the blocks were not used for training the model but were used during the testing phase. We computed 17 features from each 2-second block of sensor data. Similar to prior reports, the features were mean, variance, maximum and minimum of each accelerometer axis, Shannon entropy and mean, variance, and maximum and minimum of the Euclidean norm of the three accelerometer axes (Lum et al., 2020). We built a separate model for each limb, using stratified 5-fold cross-validation testing. We used a random forest classifier because in prior work, this approach yielded the highest overall accuracy. Importantly, when calculating accuracy and %functional use ratio, the classifier was applied to data that are not part of the training set used to train the model, simulating the case where a model trained on labeled data collected in the lab can be applied to accelerometry data collected in the home and community. Machine learning-estimated use ratios are referred to as the estimated use ratios*.*

For each random forest classifier, we calculated several performance metrics assuming functional use as the positive class and non-functional as the negative class. Accuracy is the ratio of correct classifications to total cases. Sensitivity is the ratio of true-positive classifications to all positive cases. Specificity is the ratio of true-negative classifications to all negative cases.

#### 2.5.3 Data analysis

We calculated the accuracy of machine learning-estimated use-ratio variables with respect to the ground-truth use ratios. Concurrent validity was assessed between estimated use ratios and ARAT in all participants (*n* = 31) using Pearson’s correlation coefficients. UEFM, nine-hole peg test, and MAL scores were available in a subset of the sample (*n* = 21); these were used to evaluate the validity of the estimated use ratios with respect to impairment, manual dexterity, and self-reported UE use, respectively. An *r* value ≥ 0.7 was considered a high correlation (Portney and Watkins, 2009).

To evaluate if our accelerometry approach measures the same construct as the clinical scales, factor analysis was applied to UE clinical measures and accelerometry data. A principal component analysis with varimax factor rotation was applied to ARAT, UEFM, nine-hole peg test, MAL scores, video-labeled accelerometry use ratio, and machine learning-estimated use ratios. Factors were retained if the eigenvalue exceeded 1. We also redefined the factor analysis to extract at least two factors irrespective of the eigenvalues to test if accelerometry measures are still separated from the clinical measures in an independent component.

Bland–Altman plots were used to estimate the degree of agreement between use ratios calculated with the machine learning algorithms *versus* video-labeled use ratios. Bland–Altman is a quantitative method to evaluate the agreement between two different approaches to measure the same construct. We calculated the mean difference between use ratios calculated by machine learning or video-labeled data (bias) and the standard deviation of the difference (random fluctuations around this mean difference). In addition, we computed the limits of agreement between methods as the 95% confidence intervals around the mean difference.

## 3 Results

Demographic, clinical, and UE activity characteristics of the participants are shown in Table 1. We enrolled 31 individuals (22 male, mean age ± SD = 60.38 ± 11.94 year, range = 32–83 year) with ischemic or hemorrhagic stroke (mean time since stroke = 23.8 ± 22.5 months). Participants showed moderate UE impairment, with mean ARAT ± SD = 28.96 ± 14.41, UEFM = 41.8 ± 9.4, and nine-hole peg test average time (sec) = 166.2 ± 117.5 s. Average MAL scores = 1.67 ± 1.07.

**TABLE 1:** Stroke participant demographic, stroke-related, and impairment data.

pID	Age (years)	Sex	Race	Affected limb	Handedness	Edinburgh handedness (pre-stroke)	Time post stroke (mo)	ARAT (affected)	MAL
1	64	Male	African American	Right	Left	−70	11	32	1.268
2	56	Female	African American	Left	Left	−100	25.9	38	1.018
3	71	Male	African American	Right	Right	100	14.8	53	3.357
4	77	Female	African American	Right	Right	100	13.6	7	0.75
5	65	Male	African American	Left	Right	100	17.6	27	1.071
6	54	Female	White	Right	Right	100	24.1	23	1.44
7	32	Female	Asian	Left	Right	70	6.1	6	0.464
8	53	Male	African American	Right	Right	70	12.3	29	1.179
9	50	Male	White	Right	Right	90	36.5	5	0.304
10	83	Female	African American	Left	Left	80	6.5	48	1.038
11	64	Male	African American	Right	Right	80	14	42	2.881
12	57	Male	White	Left	Right	100	17.7	32	1.32
13	78	Male	African American	Left	Right	80	18.5	52	2.571
14	66	Male	African American	Left	Right	50	13.73		0.821
15	77	Male	African American	Left	Right	90	10.1	54	3.946
16	64	Female	African American	Left	Right	100	7.2	24	2.167
17	64	Male	African American	Right	Right	100	8.1		2.893
18	48	Male	African American	Left	Right	70	9.2	47	2.786
19	77	Male	African American	Right	Right		23	41	
20	35	Male	White	Left	Right		35	23	
21	56	Male	African American	Left	Right		17	19	
22	49	Female	African American	Left	Right		19	20	
23	57	Male	White	Right	Right		104	16	
24	63	Male	White	Right	Right		77	32	
25	47	Female	African American	Right	Right		12	33	
26	50	Male	African American	Right	Right		53	15	
27	66	Male	African American	Right	Right		69	5	
28	65	Male	White	Right	Right		20	42	
29	66	Male	American Indian or Alaskan Native	Left	Right	−50	13.7	12	0.82
30	68	Female	African American	Left	Right	−100	13.9	34	2.36
31	57	Male	White	Right	Right	−100	17.03	23	1.38

Table 1. Participant characteristics. Empty cells indicate missing data.

### 3.1 Classification performance

The performance metrics for the classifier can be found in Supplementary Table S2. Average accuracy (SD) was 90.9% (4.8) in the paretic limb and 94.6% (6.2) in the less-affected limb. Sensitivity was 90.2% (6.5) in the paretic limb and 96% (4.4) in the less-affected limb. Specificity was 89.8% (6.0) and 91.9% (9.9). The video-based and estimated use ratios can be found in Supplementary Table S3. The 95% confidence interval for error in the estimated use ratio was (0.32%–2.26%), indicating that the model, on average, slightly underestimates the functional use ratio.

### 3.2 Validity of machine learning use ratio

Table 2 shows Pearson’s correlation between machine learning-estimated use ratio with corresponding measures from video-labeled ground truth data, ARAT, UEFM, nine-hole peg test, and MAL. Machine learning-estimated use ratio was significantly correlated with video-labeled use ratio (*r* = 0.99, *p* < 0.001), ARAT (*r* = 0.82, *p* < 0.001), UEFM (*r* = 0.77, *p* < 0.001), nine-hole peg test (*r* = −0.77, *p* < 0.001), and MAL (*r* = 0.61, *p* = 0.001).

**TABLE 2:** Pearson’s correlations between clinical measures and UE use ratio.

Concurrent validity against (clinical or ground-truth variable)	Domain	Machine learning estimated use ratio (95% CI)
Video-labeled (N = 31)	Ground truth	0.99, *p* < 0.001, (0.99–0.99)
ARAT (N = 31)	Prehension function	0.82, *p* < 0.001, (0.66–0.91)
UEFM (N = 21)	UE impairment	0.77, *p* < 0.001, (0.51–0.90)
Nine-hole peg test (N = 20)	Manual dexterity	−0.77, *p* = 0.001, (−0.91 to −0.49)
Motor Activity Log (N = 21)	Self-reported use	0.61, *p* = 0.001, (0.31–0.85)

Table 2. Pearson’s correlation between use ratios from video-labeled or machine learning estimates with paretic UE Action Research Arm Test (ARAT), paretic side upper extremity Fugl-Meyer (UEFM), and Motor Activity Log (MAL). The nine-hole peg test correlations are negative because the data are recorded in seconds taken to complete the task, inversely correlated with ARAT, UEFM, and use ratio. Numbers in parentheses are the 95% confidence intervals. N indicates the number of cases included in each pairwise correlation.

**FIGURE 2:**
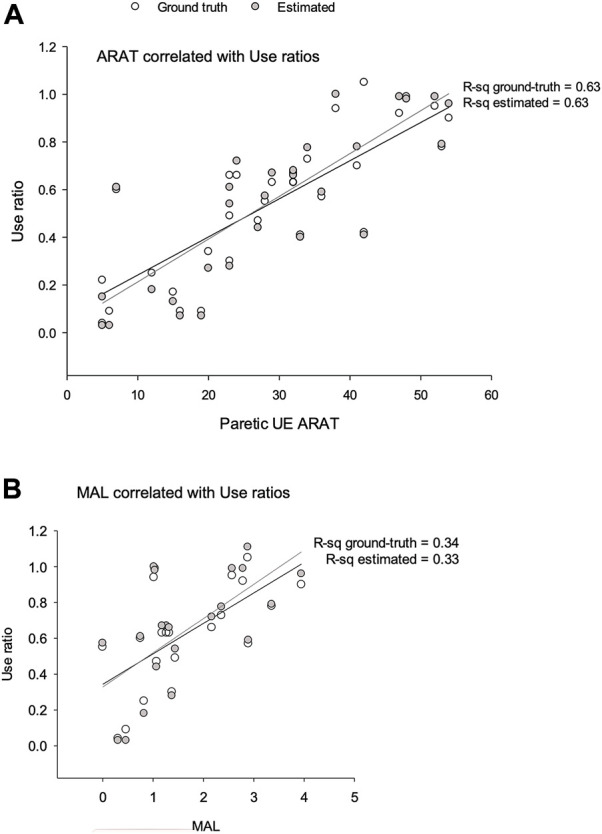
Scatter plots of video-labeled or estimated use ratios with **(A)** ARAT and **(B)** MAL.

Figure 2 shows scatter plots of video-labeled or machine learning estimates of UE use ratio with ARAT (Figure 2A) and MAL (Figure 2B). ARAT and use ratios showed a significant linear line of best fit (*R* ^2^ = 0.63). MAL showed a significant linear line of best fit with *R* ^2^ = 0.34.

A factor analysis was applied to UE behavioral measures to determine if use ratios measured the same construct as the clinical scales. We found a single factor with eigenvalues >1 and high factor loadings on the MAL, UEFM, ARAT, nine-hole peg test, and use ratios; this component explained 83% of the variance in UE motor performance. We also tested if redefining the factor analysis to extract two factors leads to use ratios splitting from the clinical scales given in our prior report (Barth et al., 2020). Table 3 shows varimax rotated factor loadings from 1- or 2-component factor solutions. The 2-factor solution accounted for 94% of the variability in UE behavioral outcomes, with use ratios, UEFM, and ARAT showing high factor loadings on component 1. MAL and nine-hole peg test showed high loading on a second maximally independent component.

**TABLE 3:** Principal component loadings for 1- and 2-factor solutions.

Variable	One-factor solution	Forced two-factor solution	
	Component 1	Component 1	Component 2
MAL	0.831	0.330	**0.910**
UEFM	0.916	**0.748**	0.533
ARAT	0.944	**0.731**	0.597
Nine-hole peg test	−0.887	−0.557	**−0.721**
Use ratio	0.940	**0.912**	0.370
Estimated use ratio	0.943	**0.911**	0.374

Table 3. Principal component and varimax rotated factor loadings for 1- and 2-factor solutions. High factor loadings (large contribution >0.6) (Reyment, 1996) are shown in bold numbers.

**FIGURE 3:**
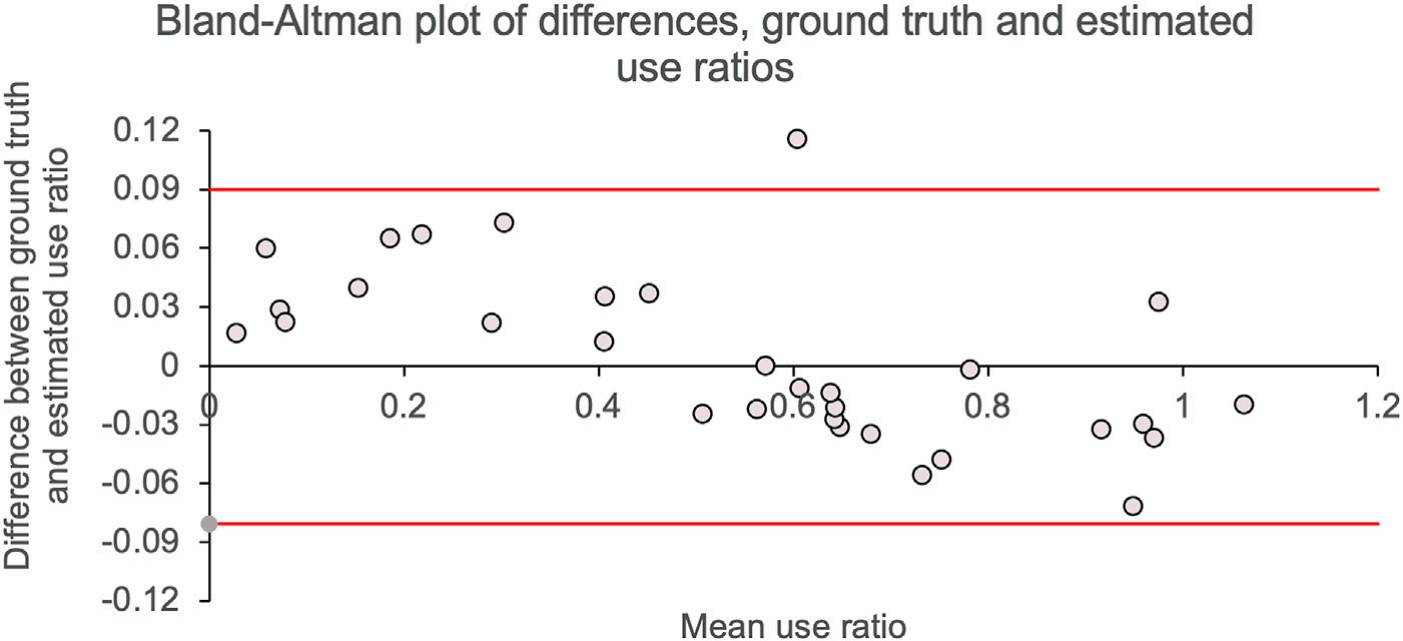
Bland–Altman plots. Difference in use ratios calculated using machine learning *versus* video-labeled accelerometry data are shown on the *y*-axis. The mean of use ratios calculated using machine learning and video-labeled accelerometry are shown on the *x*-axis. Solid gray line shows mean difference (mean ± SD = 0.004 ± 0.0.04) between use ratios calculated by the two methods; solid red lines show the 95% CI for the mean difference.

The Bland–Altman plot (Bland and Altman, 1986) quantifies the agreement between two variables measuring the same construct. The Bland–Altman plot in Figure 3 shows, for each individual, the difference between video-labeled *versus* machine learning use ratios [mean difference ± SD = 0.004 ± 0.04 (95% CI = 0.08, −0.08)] as a function of the average use ratio calculated with two methods (0.54 ± 0.31). All but one data points fall inside the Bland–Altman limits of agreement, which suggests an excellent agreement between video-labeled and machine learning estimates of use ratio.

## 4 Discussion

Machine learning predictions of functional UE use were highly correlated with video-based annotations (*r* = .99). This demonstrates that a classifier based on accelerometry features can very accurately detect periods of functional limb use in participants across a wide range of UE impairments. The amount of paretic limb functional use correlated significantly with clinical scales of impairment (Fugl-Meyer), function (ARAT), and manual dexterity (nine-hole peg test with r values between 0.77 and 0.82), while correlations with the MAL were lower (*r* = 0.61) but still significant. These results confirm the validity of the accelerometry method against existing clinical scales. The high correlations with clinical function scales and accuracy against ground-truth video annotation reflect the improvement our approach brings to accelerometry: accurate quantification of functional UE use in chronic stroke with a relatively small burden of frame-by-frame video labeling.

In prior work, we tested the performance of machine learning algorithms that separate functional from non-functional periods in accelerometry data using video annotation as ground truth and compared machine learning classifiers with the conventional count thresholding method. In this study, we applied the best-performing machine learning classifier to a larger sample of stroke participants that spanned the full impairment range and tested for concurrent validity against other clinical scales. In previously reported work, we demonstrated the feasibility of using a single wrist-worn accelerometer to detect periods of functional arm activity in a sample of 10 severe-moderately impaired stroke subjects and 10 controls (Bochniewicz et al., 2017). Using a random forest classifier, subject-specific models had accuracies of 94.8% in controls and 88.4% in the affected limb of stroke participants. In leave-one-out modeling, accuracies were 91.5% in controls and 70.2% in stroke patients. In a follow-up study, we analyzed data from the less-affected limb and the paretic limb in order to calculate ratio metrics, explored a variety of different classifiers, features, and epoch lengths (1–5 s), and compared machine learning to the conventional counts thresholding method for detecting functional use (Tran et al., 2018; Lum et al., 2020). We found that the best-performing model was random forest, which had subject-specific modeling accuracies of 96.1% and 92.6% in controls and stroke subjects, respectively. Accuracy in the dominant limb of controls and less-affected limb of stroke subjects was higher at 96.6% and 94.6%, respectively. Importantly, when calculating the functional use ratio between paretic and less-affected limbs, the conventional count method dramatically overestimated the ratio when compared to video-based ground truth. Subsequent analysis showed this overestimation was caused by the misclassification of non-functional limb movements (whole-body movements) that exceeded the threshold in the count method. In contrast, functional use ratios based on machine learning were highly accurate, with correlations of *r* = 99 with ground truth. In this study, we only focused on subject-specific modeling and expanded the sample size from 10 to 31 stroke participants spanning the full range of UE impairment. With this larger sample, the random forest classifier accuracies were comparable to prior reports, at 94.6% in the less-affected limb and 90.9% in the paretic limb. The functional use ratio continues to be highly correlated with ground truth (*r* = 99). This larger sample allowed concurrent validity testing, comparing the functional use ratio to clinical scales. We found significant correlations with tests of impairment (FM), function (ARAT), and self-reported amount of functional use (MAL), establishing concurrent validity.

Our results are consistent with prior studies reporting significant correlations between accelerometry-based metrics and clinical scales. A recent review paper of 34 studies reported a wide range of correlations between accelerometry and the MAL (0.31 < *r* < 0.84) and between accelerometry and the ARAT (0.15 < *r* < 0.79) (Heye et al., 2022). Our correlations are at the high end of the ARAT range reported by this review and near the middle of the range for the MAL. The large variability in these reported *r* values is concerning, especially for the MAL, which one would expect to have the strongest correlation with accelerometry. The large variability in prior studies could be due in part to the accelerometers responding to whole-body movements that do not incorporate functional use of the paretic limb. We previously found that the count method grossly overestimates the duration of functional UE movement compared to the ground truth based on video annotation (McLeod et al., 2016). This was due to the movement of the wrist-mounted accelerometers resulting from whole-body movements, such as ambulation. Normalizing by values from the opposite limb did not improve the estimate. Contamination of the accelerometer measurement from whole-body movements was also reported by Regterschot et al. (2021), and some methods rely on a third accelerometer on the thigh to eliminate periods of ambulation (Heye et al., 2022). Importantly, our machine learning approach overcomes these limitations by training a model that rejects accelerometer patterns from whole-body movements and only specifically detects periods of functional limb use during activities and instrumental activities of daily living.

A recent publication by Pohl et al. (2022) separated functional from non-functional movements during ADL performance in the home in individuals with stroke. They compared conventional count thresholding, optimal thresholding, and a logistic regression classifier applied to multiple IMU sensor signals. They report classification accuracy of around 80% when using an optimal threshold (>20.1 and >38.6 counts for the affected and less-affected sides, respectively). The machine learning classifier achieved similar accuracy in leave-one-out testing. Both these methods were found to be superior to the conventional thresholding method (>2 counts). Their optimal thresholding method can significantly increase the accuracy of metrics targeting the amount of functional limb use by removing slow movements that are not likely functional in nature and has the advantage of easy implementation on already collected data sets. One important difference between Pohl and our study is that their study is testing the performance of several classification schemes against video annotations. We also report the performance of our random forest classifier against video annotation ground truth, but the main purpose of our study is testing concurrent validity, correlating our results with several clinical scales. The Pohl study notes that in future work, concurrent validity with benchmark clinical outcome measures is needed. An important technical difference between studies is that their non-functional category includes minimal motion, while a third category of whole-body movements (gait, transfers, etc.) was excluded from the analysis. They note that detection and removal of whole-body movements in a pre-processing step might be needed before applying their optimal thresholding scheme; otherwise, these movements might be misclassified as functional. In contrast, our non-functional class includes minimal or no movement, and arm movements resulting from whole-body movements. So our classifier is already attempting to classify whole-body movements as non-functional, based on sensor data, and another level of pre-processing is not needed. Future work is needed to determine which approach is superior.

**FIGURE 4:**
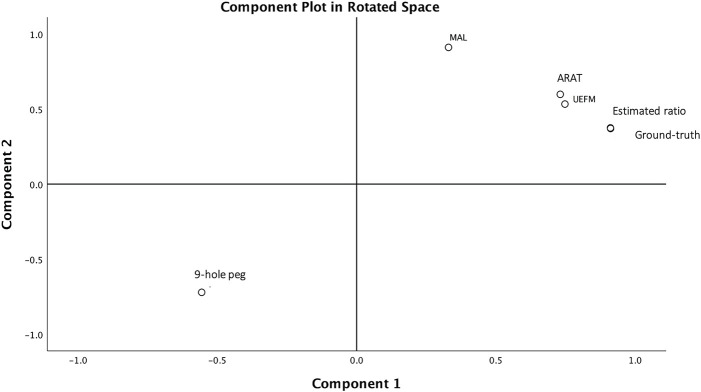
Component plot in rotated factor space. A single component accounts for 83% of the variance in UE behavioral measures of UEFM, ARAT, nine-hole peg test, self-reported MAL, and the use ratios. This is evidenced on the component plot. Forcing a 2-component solution results in MAL and nine-hole peg test scores splitting from the UEFM, ARAT, and use ratios, evidenced by MAL falling closer to component 1 axis, as shown here.

Our prior report on accelerometry outcomes within the first week of stroke showed conventionally used accelerometry counts, and the clinical scales (UEFM/ARAT) fall along two independent axes reflecting “quantity” (use ratio) *versus* “quality” (UEFM/ARAT) of movement (Barth et al., 2020). In the current report, factor analysis showed a single construct containing UEFM, ARAT, nine-hole peg test scores, MAL, and estimated use ratios, which captured 83% of the variance in impaired limb activity. Thus, the estimated use ratio captures a similar construct as the clinical scales, unlike the counts approach. Figure 4 shows the component plots from the 2-factor solution: MAL is relatively closer to component 2 in the rotated factor space, whereas the use ratio is closer to component 1. ARAT and UEFM fall along the midline between components 1 and 2, whereas the nine-hole peg test mirrors ARAT/UEFM scores along the negative axes being inversely correlated with ARAT and UEFM. Thus, the forced 2-factor solutions suggest differences in measurement properties of the MAL compared to functional UE use, and further investigation is warranted.

In our data, more than 37% of the variance in paretic limb use was not explained by ARAT scores (R-sq = 0.63). Importantly, examination of the scatter plots in Figure 2A shows that participants with similar ARAT scores can have very different paretic limb use patterns. For example, four subjects had ARAT scores between 38 and 42, but had a paretic limb use ratio between 0.42 and 1.1. Similarly, six subjects had functional use scores between 0.57 and 0.63, and ARAT scores that ranged from 7 to 36. These differences between ARAT and accelerometry, despite them measuring the same construct (as indicated by the factor analysis in present report), may result from the differing resolutions of measurement provided by accelerometry vs ARAT. Additionally, in ARAT, subtasks of varying difficulties are graded on a 0-1-2 Likert scale, and a sum score is created by simple summation assuming a 1-unit increase on easier and more difficult items representing the same amount of recovery. This approach, at least in the UEFM leads to significant measurement errors (Geed et al., 2020). The discrepancy between clinical scales and the range of actual UE use is particularly problematic for clinical studies that use neuroimaging or neurophysiology with ARAT/UEFM to understand the mechanisms of post-stroke recovery. If commonly used clinical scales do not capture true UE use, results from neurophysiology may be confounded by using only the ARAT or UEFM as the proxy for recovery.

### 4.1 Limitations

These data were collected in a simulated apartment during outpatient rehabilitation. Our next step is to acquire 24-h accelerometry data from individuals with stroke living in the community who engage in the full spectrum of ADLs/IADLs to better validate our machine learning methods for measurement of UE motor function post-stroke. In terms of the potential adoption of this method, the need for video annotation to train subject-specific classifiers limits the applicability of this method to clinical practice. However, use in clinical trials is possible, as the data collection only takes around 30 min, and a trained annotator can complete an activity script in about 2.3 h. We are currently investigating a generalizable model that can be applied to new participant data without the need for subject-specific video annotation. This may be possible if the sample size can be further increased.

## 5 Conclusion

We validated an approach to monitor long periods of functional arm use *via* accelerometers and using a machine learning classifier trained on a short period of annotated video. Our results demonstrate the feasibility of this method for the measurement of UE motor recovery in stroke rehabilitation trials.

## Data Availability

The raw data supporting the conclusion of this article will be made available by the authors, without undue reservation.
