# Supplementary material for: Concurrent validity of machine learning-classified functional upper extremity use from accelerometry in chronic stroke
Source: Front Physiol. 2023 Mar 22;14:1116878. doi: 10.3389/fphys.2023.1116878 (PMC10073694; doi:10.3389/fphys.2023.1116878)
Supplement: Supplementary file 1 [file Table1.DOCX]

Supplementary Material

Article Title

**Concurrent Validity of Machine Learning Classified Functional Upper Extremity Use from Accelerometry in Chronic Stroke**

Shashwati Geed, PT PhD^1,2^; Megan L Grainger, BS^2^; Abigail Mitchell, OTR/L^2^; Cassidy C Anderson, BS^2^; Henrike L Schmaulfuss, BS^3^; Seraphina A Culp^3^; Eilis R McCormick^3^; Maureen E McGarry, BS^3^; Mystee N Delgado, BS^3^; Allysa D Noccioli, BS^3^; Julia Shelepov^3^; Alexander W. Dromerick, MD;^1,2^ Peter S Lum, PhD^2,3^

^1^ Department of Rehabilitation Medicine, Georgetown University, Washington DC, USA

^2^ MedStar National Rehabilitation Hospital, Washington, DC, USA

^3^ Department of Biomedical Engineering, The Catholic University of America, Washington DC, USA

Supplementary Table 1. Activity Script

|  | **Activity** | **Description** |
| --- | --- | --- |
| IADLs | Laundry Task | - Carry laundry basket full of clothes to washer/dryer. Place clothes in washer/dryer and then remove. - Carry basket to closet. Hang and fold clothing items. |
|  | Linen Management | - Retrieve two pillowcases from cabinet, place pillowcases on pillow. - Simulate making the bed by pulling blanket/sheet from foot of bed up to head of bed. - Fold (2) bath towels, (2) wash cloths |
|  | Grocery Shopping | - Gather items from market shelves (one from each height) and place into a box. Carry box to car and place box into trunk. Retrieve box from trunk and place items back on shelf |
|  | Walking | - Walk up & down 20-foot hallway x3. |
|  | Kitchen Task/Meal Prep  **Note: If participants have any diet restrictions/allergies they will not consume anything.* | - Sweep trash from floor, place in trash bag, and tie trash bag. - Take food out of the refrigerator (bread and juice). Remove bread from packaging, cut a slice of bread with plastic knife. Pour juice from container into glass and take a few sips*. - Rolling out pizza dough (OT putty will be used) using rolling pin. |
|  | Financial Management/Simulated Work | - Sort through form letters from a file folder. Place letters into proper recipient envelope, place “stamp” on each envelope, address one envelope and seal both envelopes. |
|  | Medication Management | - Provided with 3 simulated prescription bottles. Pt required to open easy open lids and organize weekly pill containers based on times medications should be taken |
|  | Typing | - Type the provided 25 to 50-word passage |

List of activities and instrumental activities daily living used in the Activity Script to train the machine learning models.

Supplementary Table 2. Classifier performance: Accuracy, F1, Sensitivity, Specificity in Paretic and Less-affected arm models.

| **Supplementary Table I** | Paretic Arm Model | | | |  | Less affected arm model | | | |  |
| --- | --- | --- | --- | --- | --- | --- | --- | --- | --- | --- |
| subject | Accuracy | F1 | Sensitivity | Specificity | | Accuracy | F1 | Sensitivity | Specificity | |
| 1 | 0.90 | 0.91 | 0.94 | 0.85 | | 0.95 | 0.96 | 0.95 | 0.94 | |
| 2 | 0.73 | 0.70 | 0.67 | 0.80 | | 0.78 | 0.91 | 0.85 | 0.62 | |
| 3 | 0.92 | 0.88 | 0.88 | 0.94 | | 0.67 | 0.88 | 0.76 | 0.50 | |
| 4 | 0.87 | 0.80 | 0.77 | 0.93 | | 0.94 | 0.92 | 0.96 | 0.91 | |
| 5 | 0.98 | 0.97 | 0.96 | 0.99 | | 0.95 | 0.93 | 0.96 | 0.93 | |
| 6 | 0.85 | 0.87 | 0.89 | 0.80 | | 0.97 | 0.94 | 0.97 | 0.95 | |
| 7 | 0.93 | 0.90 | 0.90 | 0.94 | | 0.99 | 0.95 | 0.99 | 0.98 | |
| 8 | 0.96 | 0.94 | 0.93 | 0.97 | | 0.95 | 0.95 | 0.98 | 0.91 | |
| 9 | 0.91 | 0.86 | 0.81 | 0.96 | | 0.98 | 0.95 | 0.98 | 0.98 | |
| 10 | 0.91 | 0.87 | 0.86 | 0.93 | | 0.97 | 0.95 | 0.98 | 0.96 | |
| 11 | 0.86 | 0.84 | 0.85 | 0.88 | | 0.96 | 0.95 | 0.97 | 0.94 | |
| 12 | 0.90 | 0.93 | 0.94 | 0.83 | | 0.95 | 0.95 | 0.97 | 0.91 | |
| 13 | 0.94 | 0.95 | 0.96 | 0.89 | | 0.93 | 0.95 | 0.95 | 0.88 | |
| 14 | 0.97 | 0.98 | 0.98 | 0.95 | | 0.95 | 0.95 | 0.97 | 0.91 | |
| 15 | 0.95 | 0.96 | 0.97 | 0.92 | | 0.95 | 0.95 | 0.95 | 0.94 | |
| 16 | 0.91 | 0.93 | 0.93 | 0.85 | | 0.96 | 0.96 | 0.96 | 0.96 | |
| 17 | 0.93 | 0.90 | 0.88 | 0.96 | | 0.96 | 0.96 | 0.98 | 0.91 | |
| 18 | 0.90 | 0.88 | 0.88 | 0.89 | | 0.97 | 0.96 | 0.97 | 0.95 | |
| 19 | 0.95 | 0.96 | 0.97 | 0.90 | | 0.97 | 0.96 | 0.98 | 0.96 | |
| 20 | 0.87 | 0.88 | 0.90 | 0.83 | | 0.97 | 0.96 | 0.98 | 0.95 | |
| 21 | 0.87 | 0.88 | 0.90 | 0.83 | | 0.97 | 0.96 | 0.97 | 0.98 | |
| 22 | 0.98 | 0.97 | 0.97 | 0.99 | | 0.97 | 0.96 | 0.98 | 0.94 | |
| 23 | 0.93 | 0.95 | 0.97 | 0.88 | | 0.97 | 0.96 | 0.97 | 0.96 | |
| 24 | 0.87 | 0.88 | 0.90 | 0.77 | | 0.95 | 0.96 | 0.95 | 0.93 | |
| 25 | 0.96 | 0.93 | 0.94 | 0.96 | | 0.96 | 0.96 | 0.97 | 0.94 | |
| 26 | 0.87 | 0.88 | 0.89 | 0.84 | | 0.96 | 0.96 | 0.98 | 0.94 | |
| 27 | 0.90 | 0.91 | 0.92 | 0.89 | | 0.98 | 0.96 | 0.98 | 0.98 | |
| 28 | 0.92 | 0.91 | 0.92 | 0.91 | | 0.98 | 0.96 | 0.98 | 0.97 | |
| 29 | 0.92 | 0.88 | 0.87 | 0.95 | | 0.97 | 0.96 | 0.97 | 0.96 | |
| 30 | 0.90 | 0.92 | 0.93 | 0.85 | | 0.97 | 0.96 | 0.98 | 0.95 | |
| 31 | 0.93 | 0.90 | 0.89 | 0.95 | | 0.95 | 0.96 | 0.97 | 0.91 | |
|  |  |  |  |  | |  |  |  |  | |
| average | 0.91 | 0.90 | 0.90 | 0.90 | | 0.95 | 0.95 | 0.96 | 0.92 | |
| sd | 0.05 | 0.06 | 0.06 | 0.06 | | 0.06 | 0.02 | 0.04 | 0.10 | |

Supplementary Table 3. Video-based and estimated use and use ratios.

|  | Paretic functional use | |  | Less Affected functional use | |  | Use ratios | | |
| --- | --- | --- | --- | --- | --- | --- | --- | --- | --- |
| subject | Video | Estimated |  | Video | Estimated |  | Video | Estimated | error |
| 1 | 0.549 | 0.582 |  | 0.780 | 0.754 |  | 0.704 | 0.772 | -0.067 |
| 2 | 0.465 | 0.415 |  | 0.702 | 0.736 |  | 0.662 | 0.564 | 0.098 |
| 3 | 0.058 | 0.056 |  | 0.677 | 0.694 |  | 0.086 | 0.081 | 0.005 |
| 4 | 0.285 | 0.250 |  | 0.840 | 0.849 |  | 0.340 | 0.294 | 0.045 |
| 5 | 0.070 | 0.067 |  | 0.786 | 0.794 |  | 0.089 | 0.085 | 0.004 |
| 6 | 0.550 | 0.589 |  | 0.870 | 0.864 |  | 0.632 | 0.681 | -0.049 |
| 7 | 0.363 | 0.368 |  | 0.882 | 0.897 |  | 0.411 | 0.410 | 0.001 |
| 8 | 0.136 | 0.134 |  | 0.790 | 0.825 |  | 0.172 | 0.163 | 0.009 |
| 9 | 0.203 | 0.179 |  | 0.932 | 0.941 |  | 0.218 | 0.191 | 0.027 |
| 10 | 0.267 | 0.268 |  | 0.629 | 0.637 |  | 0.424 | 0.420 | 0.004 |
| 11 | 0.433 | 0.464 |  | 0.876 | 0.902 |  | 0.494 | 0.515 | -0.021 |
| 12 | 0.750 | 0.793 |  | 0.794 | 0.820 |  | 0.944 | 0.966 | -0.022 |
| 13 | 0.721 | 0.747 |  | 0.685 | 0.703 |  | 1.053 | 1.063 | -0.010 |
| 14 | 0.727 | 0.740 |  | 0.733 | 0.757 |  | 0.991 | 0.978 | 0.013 |
| 15 | 0.761 | 0.819 |  | 0.834 | 0.839 |  | 0.913 | 0.976 | -0.063 |
| 16 | 0.727 | 0.740 |  | 0.932 | 0.922 |  | 0.781 | 0.803 | -0.023 |
| 17 | 0.372 | 0.356 |  | 0.791 | 0.818 |  | 0.470 | 0.435 | 0.035 |
| 18 | 0.460 | 0.486 |  | 0.731 | 0.742 |  | 0.629 | 0.655 | -0.026 |
| 19 | 0.781 | 0.815 |  | 0.820 | 0.829 |  | 0.952 | 0.983 | -0.031 |
| 20 | 0.535 | 0.572 |  | 0.847 | 0.860 |  | 0.632 | 0.665 | -0.034 |
| 21 | 0.545 | 0.580 |  | 0.863 | 0.867 |  | 0.631 | 0.669 | -0.038 |
| 22 | 0.030 | 0.028 |  | 0.821 | 0.838 |  | 0.037 | 0.033 | 0.003 |
| 23 | 0.750 | 0.793 |  | 0.834 | 0.845 |  | 0.900 | 0.939 | -0.039 |
| 24 | 0.590 | 0.667 |  | 0.891 | 0.891 |  | 0.662 | 0.748 | -0.086 |
| 25 | 0.076 | 0.060 |  | 0.866 | 0.872 |  | 0.088 | 0.069 | 0.019 |
| 26 | 0.530 | 0.558 |  | 0.883 | 0.896 |  | 0.601 | 0.623 | -0.022 |
| 27 | 0.501 | 0.517 |  | 0.877 | 0.877 |  | 0.571 | 0.590 | -0.019 |
| 28 | 0.436 | 0.459 |  | 0.791 | 0.807 |  | 0.551 | 0.569 | -0.018 |
| 29 | 0.203 | 0.180 |  | 0.806 | 0.809 |  | 0.251 | 0.222 | 0.029 |
| 30 | 0.601 | 0.638 |  | 0.826 | 0.839 |  | 0.728 | 0.761 | -0.034 |
| 31 | 0.252 | 0.249 |  | 0.840 | 0.853 |  | 0.301 | 0.292 | 0.008 |
|  |  |  |  |  |  |  |  |  |  |
| Average |  |  |  |  |  |  | 54.57% | 55.54% | -0.97% |
| SD |  |  |  |  |  |  | 29.32% | 31.10% | 3.67% |
|  |  |  |  |  |  |  | confidence | 0.32% | -2.26% |
